# Greenspace, stress, and health: how is epigenetics involved?

**DOI:** 10.3389/fpubh.2024.1333737

**Published:** 2024-02-16

**Authors:** Ugoji Nwanaji-Enwerem, John E. McGeary, Diana S. Grigsby-Toussaint

**Affiliations:** ^1^Department of Behavioral and Social Sciences, Brown University School of Public Health, Warren Alpert Medical School of Brown University, Providence, RI, United States; ^2^Providence VA Medical Center, Providence, RI, United States; ^3^Department of Psychiatry and Human Behavior, Warren Alpert Medical School of Brown University, Providence, RI, United States; ^4^Department of Behavioral and Social Sciences, Department of Epidemiology, Center for Health Promotion and Health Equity, Brown University School of Public Health, Providence, RI, United States; ^5^Department of Behavioral and Social Sciences, Center for Health Promotion and Health Equity, Brown University School of Public Health, Providence, RI, United States

**Keywords:** green space, epigenetics, environment, stress, exposure

## Abstract

Most expositions of the association between green space and overall health and well-being focus on psychosocial mechanisms. However, discussions of the biological underpinnings of the exposure to green space and health implications are limited. In this paper, we highlight the role epigenetics plays in the manifestation or suppression of stress, in addition to some of the proposed epigenetic mechanisms through which green space mitigates stress. The Health: Epigenetics, Greenspace and Stress (HEGS) model is introduced to explicate this association, and suggestions for research to build the evidence base in this area are discussed.

## Introduction

Greenspace is a critical feature of a healthy built environment. Exposure to greenspace fosters improved wellness and health among living organisms. Defined as the density of vegetated land, greenspace includes parks and reserves, forests, grasslands, fields, greenways and trails, community gardens, street trees, and nature conservation regions, to name a few (1). Several studies have outlined the beneficial health outcomes associated with greenspace exposure including decreased mortality, enhanced sleep quality, better pregnancy outcomes, reduced risk of allergic and cardiovascular diseases, and improved general and mental health (2). From a mechanistic perspective, four key domains have been proposed as pathways that explain the relationship between greenspace and health. *Harm mitigation, capacity instoration, immunity improvement* and *capacity restoration* are discussed as underlying processes through which greenspace is linked to health (3, 4). *Harm mitigation* refers to the buffering feature of greenspace in reducing exposure to environmental pollutants such as air, noise, and heat*. Capacity instoration* refers to the role of greenspace in promoting physical activity and fostering social cohesion (3). Improved immune functioning refers to how greenspace exposure results in exposure to biodiversity that favorably improves systemic immunity (4). *Capacity restoration* refers to the effect of greenspace in renewing attention and reducing physiological stress, the latter of which will be a main focus of this paper.

While most explanations of the effect of greenspace exposure on health are grounded in psychosocial benefits, there is a need for additional empirical evidence exploring the biological mechanisms through which greenspace influences health. Further understanding the interactive nature of both biological and psychosocial mechanisms of greenspace and health would be beneficial. This paper introduces the Health: Epigenetics, Greenspace, and Stress (HEGS) conceptual model which seeks to provide greater understanding of these processes and to identify key gaps in the field regarding the epigenetic influences underlying how greenspace exposure impacts stress and health.

## Greenspace and epigenetics

Epigenetics refers to chemical modifications at the molecular level that change gene expression but do not change the DNA sequence (i.e., the genetic code) (5). Formally defined as gene expression differentiation due to epigenome wide alterations, epigenetics includes chemical modification of DNA through mechanisms such as DNA methylation and other processes such as histone modification, and microRNA alterations that result in gene expression changes (5).

DNA methylation is a molecular process used to explore underlying mechanistic pathways of environmental exposures, such as greenspace, to overall health and disease risk. The methylation process takes place when specific enzymes, called *DNA methyltransferases,* add a methyl group to a cytosine(C) base to generate 5-methylcytosine, which is usually attached to a guanine(G) base, resulting in methylation at a CpG site (5). DNA methylation may affect the expression of downstream components that stimulate disease manifestation or progression. Recent studies have suggested that this process is more plastic at certain periods of development. This age window of “increased” epigenetic plasticity has important health outcome implications about how timing of exposure to environmental stimuli such as greenspace and stress can alter methylation patterns of specific genes (5).

Jeong et al. (6) highlight the basis for the plausibility that greenspace can influence the DNA methylome, based on evidence of associations between greenspace and environmental factors (i.e., physical and chemical) shown to be associated with DNA methylation and based on studies that show that lack of greenspace in the context of neighborhood deprivation affects DNA methylation. Importantly, their epigenome wide association study (EWAS) showed that DNA methylation profiles, specifically 163 and 56 differentially methylated regions were identified as significant for residential greenness at 30 and 500 meter buffers of residential address, respectively. For allostatic load, a measure of chronic stress, 1,675 CpGs were identified from the EWAS as associated with relevant phenotypes (6). Xu et al. (7) also identified methylation differences at several CpG sites to be associated with greenspace exposure. CpG sites associated with greenspace exposure were mapped onto genes related to mental health disorders, cancers, and nutritional and metabolic diseases (7). These studies indicate the relevance of exploring epigenetic mechanisms to better understand association pathways between greenspace and health.

## Stress and epigenetics

In a constantly evolving environment, health and wellness are dependent on the ability to maintain homeostasis against actual and perceived threats (8). Stressors are defined as threats to homeostasis that can elicit a stress response. The hypothalamic–pituitary- adrenal (HPA) axis regulates stress response, adaptation, and restoration of balance after exposure to a stressor. Epigenetic modifications have been suggested as a regulatory mechanism behind how the HPA axis adapts when increased and/or chronic stress affects its normal functioning. Epigenetic modifications affect the transcription and translation of regulatory genes involved in the HPA axis which can result in neuroendocrine disarray and the manifestation of stress related disorders (8). In addition to the independent epigenetic effects of stressors on health, it is possible that some epigenetic modifications that are associated with stress overlap or are modified by epigenetic modifications associated with greenspace exposure.

## Greenspace and stress

Greenspace is an important environmental exposure related to the physiological stress response.

Greenspace exposure is also associated with benefits to psychological well-being through several pathways, including stress reduction. The functionality of greenspace exposure to reduce stress is associated with the previously discussed mechanistic domains of capacity restoration and instoration (e.g., increased physical activity and attention restoration). Roe et al. (9) show that there is a significant and inverse relationship between higher greenspace exposure levels and stress levels. In addition, this study also found that higher levels of greenspace exposure were associated with healthier cortisol levels in women and had a mitigating effect in high cortisol levels among men (9). More specifically, greenspace interventions which involve nature-based activities to increase time spent in greenspace, have also been shown to aid in stress relief, with evidence supporting greenness exposure to better regulate and maintain healthy cortisol levels (10).

## Environment and the epigenome

It is important to highlight that green space exposure coexists with other environmental exposures such as air and noise pollution (11). The interplay of these exposures collectively have health effects (3, 11). Greenspace has been shown to have a buffering capacity in reducing the adverse health effects associated with co-existing environmental hazards such as air and noise pollution, which also induce the physiological stress response (12). The epigenetic toxicity of ambient risk factors is also relevant to the scope of this paper. Epigenetic responses, including DNA methylation changes and epigenetic aging, to environmental exposures such as air pollution may occur as early as *in utero* and can have risks as severe as mortality (13, 14). Prenatal ambient air pollution exposure has been found to be associated with epigenetic aging at birth, with long term implications for health (15). Such findings further indicate the importance of time course considerations regarding other environmental exposures associated with greenspace and how epigenetic responses mediate resultant health outcomes.

**Figure 1:**
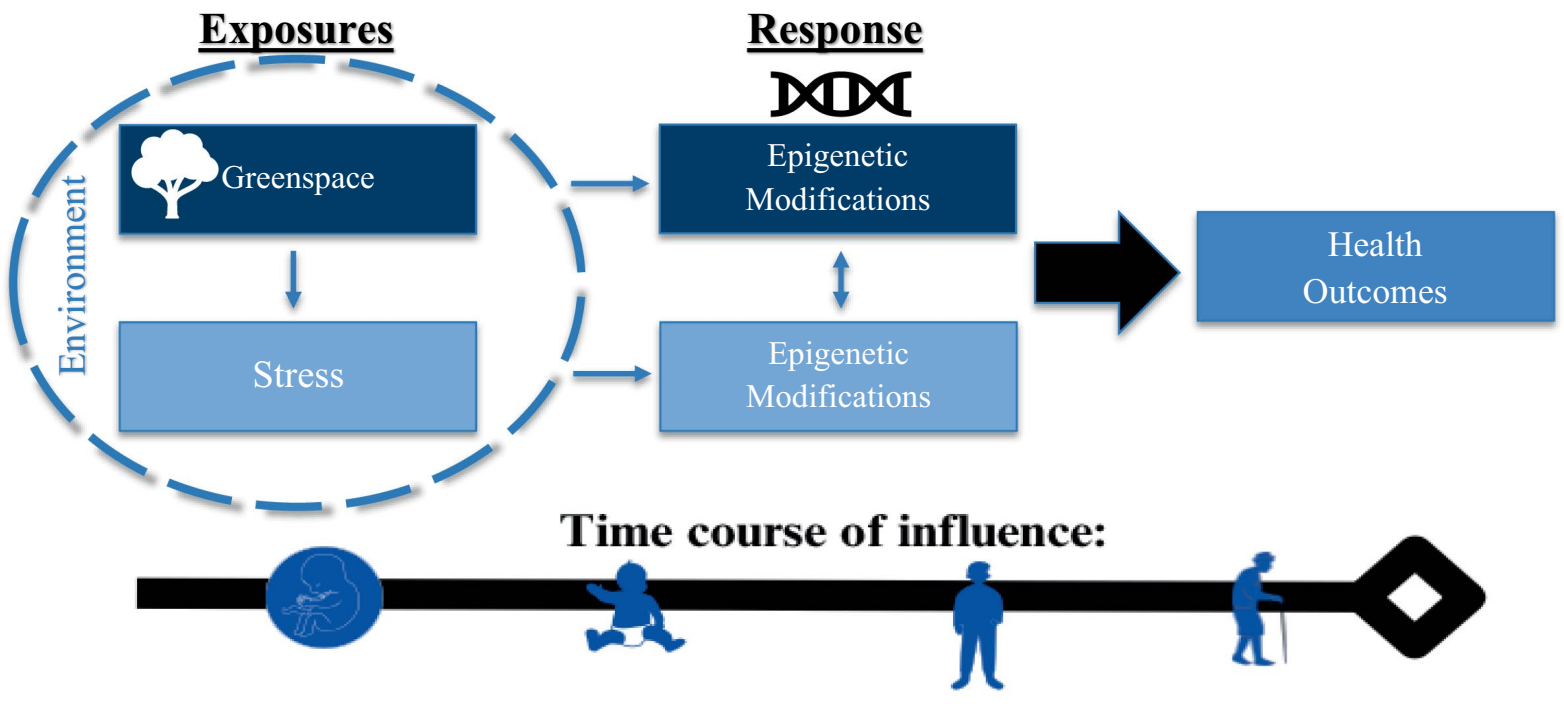
Health: epigenetics, greenspace, and stress (HEGS) conceptual model.

Based on this exploration of the literature, we propose the HEGS conceptual model which highlights epigenetic considerations that impact the pathways through which environmental exposure to greenspace affects health (see Figure 1). The conceptual model shows that greenspace exposure and stress, which exist in the sphere of the larger environment, are exposures that independently result in epigenetic modifications that result in various health outcomes. Based on research evidence, greenspace is known to attenuate effects of stress, which is indicated by the lightening of the boxes of stress and its associated epigenetic modifications. Importantly, what remains suggested yet not fully elucidated is the potential overlap or physiological interaction between epigenetic changes associated with greenspace and stress, respectively. This interaction is displayed by the bidirectional arrow between exposure-specific epigenetic modifications. Understanding if hypermethylation of a certain gene caused by a stressor becomes less methylated or vice versa based on greenspace exposure remains unknown. In essence, the nature of the co-dependency or interplay of epigenetic changes between greenspace and stress is plausible yet poorly studied.

The bottom arrow of the model labeled time course of influence highlights the fact that epigenetic modifications occur across the life course and are heritable and reversible, beginning from *in-utero* exposure through adulthood. Several studies show that maternal exposure to greenspace affects child’s health and leads to epigenetic changes in genes associated with mental health. Prenatal greenspace exposure has been associated with reduced odds of autism spectrum disorder and healthier birth weights (16, 17). Notably, maternal greenspace exposure has been shown to have a positive association with methylation status of *HTR2A* in placental tissue which has significant implications for serotonin and child neurodevelopment (18). Maternal prenatal stress results in hormone dysregulation and affects the epigenetic regulation of placental HPA axis (i.e., glucocorticoid) genes that can elevate susceptibility of psychopathology (19). Further understanding the influence of “stage of life course” and cumulative exposure of greenspace exposure on the reversibility of epigenetic changes to certain genes remains unknown in the field. Greater exploration of the compounded effect of increased vulnerability to stress, on susceptibility to greenspace induced irreversible epigenetic alterations is not understood. Thus, better understanding of whether the plasticity of these interactive epigenetic pathways occurs from time of birth into adulthood is an additional area that needs further attention. As previously highlighted, the literature reports that epigenetic mechanisms are highly active in children between ages 0 and 5 years with heightened sensitivity to environmental exposures, such as greenspace and stress, which may have important implications for early timing of interventions.

The bidirectional arrow of the HEGS conceptual model is indicative of a poorly understood pathway, such that further exploration could contribute to significant advances regarding the underlying mechanisms at play in the associations between greenspace, stress, and health.

## Discussion

Epigenetic evidence highlights the impact of the environment and more specifically environmental characteristics on health outcomes. Greenspace is a highly relevant environmental characteristic that affects all humans. Further understanding the epigenetic mediational pathways in terms of how greenspace exposure reduces stress and generates less risk for morbidity and mortality is an important area of environmental science with significant public health implications.

The reversibility of epigenetic modifications based on environmental stimuli shows the great potential of a positive exposure such as greenspace in influencing physiological control and response to negative exposures such as stress. An important reality is that the pathways through which greenspace is linked with health benefits are multiple in number and multifactorial in nature. The relative contributions of these different pathways and factors on overall health may depend on exposure to specific forms of greenspace, such as trees or grasslands or parks compared to neighborhood vegetation which may vary based on ecological properties. This highlights the need for further identification of measures that can be used to better understand the role of greenspace characteristics, such as type and quality on health (20). Among the complexity of the pathways of greenspace and health, there are clear indications that stress reduction may be the most important pathway in further understanding key epigenetic associations. Acquiring more concrete evidence explaining the following is where the field is lacking:

1. Elucidating specific methylation changes caused by greenspace exposure, such as hyper or hypomethylation, and its effect on increasing or decreasing expression of genes associated with specific diseases, with understanding that epigenetic changes are tissue specific and the relevant tissues are not known
2. Identifying the critical periods developmentally for epigenetic modification as well as how this might interact with cumulative exposure to environmental agents and their epigenetic influences
3. Ascertaining the potential overlap and interplay between stress impacts and greenspace exposure at an epigenetic level

With this understanding of the relationship between greenspace and stress, environmental epigenetics becomes relevant in answering how the environment gets under the skin physiologically. Clarity on how the epigenome responds to greenspace exposure, the extent to which these alterations directly initiate or worsen human disease, and the mechanisms through which these modifications are inherited and interact are crucial points warranting further study. Considerations of greenspace and stressors as components of the environment and their impact on health highlight opportunities for interventions to prevent diseases and improve the overall state of physiological wellness. The field of epigenetics and understanding its underpinnings in environmental science and public health informs actions (e.g. advocacy, interventions, therapeutics) to prevent poor health outcomes and improve the health of the greater population. The flexibility of the epigenome offers hope and also stimulates a multitude of additional areas for future work examining interacting psychosocial and biological roots of environmental influence on well-being. Moreover, exploration of the epigenetic mediating effects of greenspace and stress exposure on health is an exciting terrain to better understand with growing importance.

## Data availability statement

The original contributions presented in the study are included in the article/supplementary material, further inquiries can be directed to the corresponding author.

## Author contributions

UN-E: Conceptualization, Writing – original draft, Writing – review & editing, Visualization. JM: Writing – review & editing. DG-T: Conceptualization, Funding acquisition, Writing – original draft, Writing – review & editing.
